# Reservoir Competence of Wildlife Host Species for *Babesia microti*

**DOI:** 10.3201/eid1812.111392

**Published:** 2012-12

**Authors:** Michelle H. Hersh, Michael Tibbetts, Mia Strauss, Richard S. Ostfeld, Felicia Keesing

**Affiliations:** Bard College, Annandale-on-Hudson, New York, USA (M.H. Hersh, M. Tibbetts, M. Strauss, F. Keesing);; and Cary Institute of Ecosystem Studies, Millbrook, New York, USA (M.H. Hersh, R.S. Ostfeld)

**Keywords:** Babesia microti, babesiosis, bacteria, disease reservoirs, ecology, Ixodes scapularis, Peromyscus, ticks, zoonoses, real-time PCR, competence, wildlife host species

## Abstract

Human babesiosis is an increasing health concern in the northeastern United States, where the causal agent, *Babesia microti*, is spread through the bite of infected *Ixodes scapularis* ticks. We sampled 10 mammal and 4 bird species within a vertebrate host community in southeastern New York to quantify reservoir competence (mean percentage of ticks infected by an individual host) using real-time PCR. We found reservoir competence levels >17% in white-footed mice (*Peromyscus leucopus*), raccoons (*Procyon lotor*), short-tailed shrews (*Blarina brevicauda*), and eastern chipmunks (*Tamias striatus*), and <6% but >0% in all other species, including all 4 bird species. Data on the relative contributions of multiple host species to tick infection with *B. microti* and level of genetic differentiation between *B. microti* strains transmitted by different hosts will help advance understanding of the spread of human babesiosis.

## Abstract 2

Competence data will aid understanding of the spread of human babesiosis.

Human babesiosis is a growing public health concern, especially in the northeastern United States. Babesiosis is a zoonotic, malaria-like illness that can be particularly severe and sometimes fatal in elderly, asplenic, or immunocomprised persons (*1*). In the lower Hudson Valley region of New York State, 5 locally acquired cases of babesiosis were documented in 2001 (*2*), and incidence has increased 20-fold from 2001 through 2008 (*3*).

The causal agent of human babesiosis, *Babesia microti* (Apicomplexa: Piroplasmida), is a protozoan blood parasite that is transmitted in nature by the bite of an infected tick. In the northeastern United States, the vector of this disease is *Ixodes scapularis*, the black-legged tick, which is also the primary vector of *Borrelia burgdorferi* and *Anaplasma phagocytophilum,* the causal agents of Lyme disease and human granulocytic anaplasmosis, respectively. *B. microti* is not known to be transmitted transovarially on the basis of available evidence (*4*–*7*), indicating that it is not passed from infected adult ticks to eggs, and therefore must be acquired from a blood meal on an infected host. Larval ticks bite infected animals and obtain the pathogen, molt into nymphs, and overwinter. The following year, they then can infect additional hosts when seeking a second blood meal as nymphs or a third blood meal as adults. *B. microti* has been detected in questing adult and nymphal ticks in studies across the northeastern United States (*8*–*12*). In the case of Lyme disease, which is transmitted by the same vector, the nymphal stage is most relevant to human health because bites from nymphs often go undetected and provide greater opportunities to transmit the pathogen (*13*,*14*). Babesiosis can also be acquired through blood transfusions, another growing concern for public health (*15*).

*B. microti* can infect a range of animal species, but the reservoir competence of many wildlife hosts in the northeastern United States is not well known. We define reservoir competence as the mean percentage of ticks infected by any individual host of a given species. Furthermore, many studies test for the presence of or exposure to the pathogen in host blood or tissue, which provides useful information about host infection, but not information on how often infected animals transmit the pathogen to tick vectors. White-footed mice (*Peromyscus leucopus*) and meadow voles (*Microtus pennsylvanicus*) have been established as reservoir hosts on the basis of seroprevalence (*16*,*17*), and *P. leucopus* mice are known to transmit the pathogen to ticks (*18*). Other rodent species, including species in other genera of mice (*Apodemus* and *Sicista*) and voles (*Eothenomys, Lagurus,* and *Myodes*) are known hosts in Europe (*19*–*21*) and Asia (*22*–*24*). *B. microti* or *B. microti*–like infection has been observed in other common eastern US mammal species, such as short-tailed shrews (*Blarina brevicauda*) (*25*), eastern cottontail rabbits *(Sylvilagus floridanus*) (*17*), eastern chipmunks (*Tamias striatus*) (*17*), raccoons (*Procyon lotor*) (*26*) and foxes (*27*), and congeneric species in other regions or countries, including *Sciurus* spp. squirrels (*28*) and *Sorex* spp. shrews (*29*). Birds have not been extensively tested for *B. microti,* but in Europe, evidence of *B. microti* infection has recently been discovered in engorged larval ticks that had been feeding on birds of several species (*30*–*32*). Determining the role that any of these species play in *B. microti* dynamics in nature requires information on the rate at which hosts transmit *B. microti* to tick vectors.

Few systematic surveys have been conducted that compare reservoir competencies of multiple potential host species for *B. microti*. Testing a broad sample of wildlife species would enable us not only to identify which species transmit the pathogen, but also which species act as weakly competent or incompetent hosts, providing blood meals to ticks but rarely or never transmitting the pathogen (*33*). Infection of nymphs by other tickborne pathogens, such as *B. burgdorferi*, is affected by the presence of strongly and weakly competent reservoir hosts in the same communities (*34*,*35*). On Nantucket Island, an area well studied for *B. microti*, multiple hosts transmit the pathogen but differ in infection prevalence (*17*). Although several studies have extensively sampled small mammal communities for seroprevalence of *B. microti* (*19*–*23*), comprehensive surveys of more diverse host communities, for either seroprevalence or reservoir competence, are rare.

In this study, we sought to determine the level of reservoir competence for *B. microti* for as many host species as possible in a babesiosis-endemic region (Dutchess County, New York) where human babesiosis cases are rapidly increasing (*3*). We designed and tested a real-time PCR method to determine whether ticks were infected with *B. microti*. To determine the relative levels of reservoir competence in as many potential host species as possible, we applied this method to sample newly molted nymphal ticks that fed as larvae on a range of potential wildlife hosts. We tested the hypothesis that white-footed mice (*P. leucopus*) were the predominant host of *B. microti* in this community. We also compared these results to levels of prevalence in questing nymphal ticks from the same region. Our overall goal was to improve understanding of the role of multiple wildlife host species in *B. microti* transmission.

## Methods

### Field Methods

Hosts were trapped on the property of the Cary Institute of Ecosystem Studies (Millbrook, NY, USA) during the peak abundance of larval black-legged ticks (*I. scapularis*) in July–September in 2008, 2009, and 2010. Hosts included 10 mammal and 4 bird species (Table 1). We focused our sampling efforts on common forest-dwelling terrestrial mammals and ground-dwelling songbirds known to host *I. scapularis* ticks. Hosts were held for 3 days in cages with wire mesh floors suspended over pans lined with wet paper towels so that ticks could feed to repletion, drop from hosts, and be collected. Our ideal was to sample 10–25 ticks/individual host, but our ability to meet this depended on host tick loads. If hosts did not drop enough ticks within 3 days, we increased sample size when possible for selected individuals by infesting them with unfed larval ticks according to the methods of Keesing et al. (*35*).

**Table 1:** Host species tested for *Babesia microti* reservoir competence, southeastern New York, USA, 2008–2010*

Host species	Common name	No. hosts tested	No. ticks tested	Mean no. ticks sampled per host (range)
Mammals				
* Blarina brevicauda*	Northern short-tailed shrew	28	534	19.1 (12–25)
* Didelphis virginiana*	Virginia opossum	24	464	19.3 (11–25)
* Glaucomys volans*	Northern flying squirrel	5	84	16.8 (6–25)
* Mephitis mephitis*	Striped skunk	2	31	15.5 (10–21)
* Peromyscus leucopus*	White-footed mouse	17	308	18.1 (11–25)
* Procyon lotor*	Raccoon	21	396	18.9 (10–25)
* Sciurus carolinensis*	Eastern gray squirrel	18	333	18.5 (10–25)
* Sorex cinereus*	Masked shrew	6	41	6.8 (4–10)
* Tamias striatus*	Eastern chipmunk	15	245	16.3 (10–25)
* Tamiasciurus hudsonicus*	Eastern red squirrel	15	295	19.7 (11–25)
Birds				
* Catharus fuscescens*	Veery	15	310	20.7 (10–25)
* Dumetella carolinensis*	Gray catbird	13	240	18.5 (10–24)
* Hylocichla mustelina*	Wood thrush	18	318	17.7 (10–25)
* Turdus migratorius*	American robin	17	293	17.2 (8–23)

*Number of ticks tested per host can include samples from either natural tick loads or experimental infestations and are not representative of total tick loads.

Larval ticks were either collected in the field or hatched from eggs in the laboratory. Larvae hatched from eggs in the laboratory were the offspring of locally collected adult ticks fed on uninfected rabbits. Because transovarial transmission of *B. microti* is not known to occur (*4*–*7*), these infestations did not affect host exposure to the pathogen. Hosts that had been infested were held for an additional 4 days and engorged ticks were collected each day. All engorged larval ticks were held in moistened glass vials at constant temperature and humidity until they molted into the nymphal stage. Newly molted nymphs were flash-frozen in liquid nitrogen and stored at −80°C. All procedures were conducted with approval from the Cary Institute of Ecosystem Studies Institutional Animal Care and Use Committee.

To provide a context for assessing the reservoir competence of hosts at the study site, we also sampled questing nymphal ticks in June 2010 (13 sites) and June 2011 (5 sites) in the towns of Washington, New York, and adjacent Pleasant Valley, New York. We collected questing nymphal ticks and estimated nymphal density by drag sampling (*36*). Corduroy cloths (1 m^2^) were dragged along 400-m transects in each site once or twice in a given year during the annual peak in nymphal questing activity. Ticks were counted and collected every 15–30 min. Questing nymphs were flash frozen upon collection and stored as described above. All sites sampled for questing nymphs were in oak-dominated eastern deciduous forests as described by Ostfeld et al. (*36*) either on the grounds of or within 12 km of the Cary Institute of Ecosystem Studies, where trapping occurred. For each site, the total density of nymphs was multiplied by the proportion of infected nymphs to provide an estimate of the density of infected nymphs.

### DNA Extraction and Amplification

Only ticks from hosts that produced a minimum of 10 newly molted nymphs were tested for infection, with the exception of flying squirrels (*Glaucomys volans*), masked shrews (*Sorex cinereus*), and American robins (*Turdus migratorius*), which had low tick loads. For these species, we tested ticks from hosts with >4 newly molted nymphs, but considered these data provisional given low sample sizes per individual host.

To obtain DNA from the ticks, we extracted total genomic DNA using either the DNeasy Blood and Tissue Kit (QIAGEN, Hilden, Germany) or the Gentra Puregene Tissue Kit (QIAGEN). Each DNA extraction included a negative control of unfed larval ticks. We designed 2 primers to amplify a 133-bp fragment of the 18S rDNA region in the *B. microti* species complex (including all clades within *B. microti*) (*37*), smbaJF (5′-GCG TTC ATA AAA CGC AAG GAA GTG T-3′), and smbaKR (5′-TGT AAG ATT ACC CGG ACC CGA CG-3′).

We then amplified DNA in a real-time PCR by using SYBR green technology in a C1000 Thermal Cycler with CFX96 Optical Reaction Module (Bio-Rad, Hercules, CA, USA). The reaction mixture included 12.5 μL iQ SYBR Green Supermix (Bio-Rad), 1.25 μL of 10 μmol/L solutions of each primer, 7.5 μL autoclaved or filter-sterilized ultrapure water, and 2.5 μL of template (undiluted tick extracts). Reaction conditions were 10 min at 95°C, followed by 40 cycles for 10 s at 95°C, 20 s at 68°C, and 40 s at 72°C. As a positive control, we used *B. microti* isolates from human patient provided by the New York State Department of Health (Albany, NY, USA). DNA extractions from unfed larval ticks and ultrapure water were used as negative controls to account for potential contamination during the extraction and PCR. To prevent contamination between samples, barrier pipette tips were used throughout the process. Three replicate PCRs were run per tick.

We incorporated melting curve analysis after amplification to distinguish true-positive samples from false-positive samples or mispriming. PCR products were heated from 72°C to 90°C; temperature was increased by 0.5°C every 30 s. Positive controls consistently had melting point maxima of 84°C or 84.5°C. To confirm PCR results, a subset of 197 real-time PCR products were sequenced, including samples with melting point maxima close to the range of standards (83.5°C–84.5°C) and representative samples of products amplifying at different melting point maxima from different host species (Table 2). PCR products were purified by using a QIAquick PCR Purification Kit (QIAGEN) and sequenced by using an ABI 3730XL Autosequencer (Applied Biosystems, Carlsbad, CA, USA). Sequences were edited manually by using FinchTV (Geospiza, Seattle, WA, USA), and identity of sequences was confirmed by using basic local alignment search tool (BLAST) searches (National Center for Biotechnology Information, Bethesda, MD, USA) of GenBank and the blastn algorithm (*38*). Identical molecular protocols were used for analysis of questing nymphs.

**Table 2:** Sequenced PCR products with different melting point maxima for *Babesia microti* real-time PCR and primers smbaJF and smbaKR, southeastern New York, USA, 2008–2010

Melting point maximum, °C	No.* B. microti*–positive samples/no. tested
≤82	0/70
82.5	1/3
83	1/4
83.5	20/20
84	42/43
84.5	14/16
85	0/5
≥85.5	0/36

Ticks were considered positive for *B. microti* if any 1 of 3 replicate samples amplified and had a melting point maximum of 83.5°C–84.5°C. Ticks with marginal results (positive replicates with double peaks, positive replicates with a melting point maximum of 83°C, or replicates that amplified and had a melting point maximum of 83.5°C–84.5°C but low fluorescence) were run a second time. If results of the second run met the criteria for positivity described above, ticks were considered positive for *B. microti*. If results of the second run were marginal or negative, ticks were considered negative for *B. microti*. Reservoir competence for each host species was calculated as the average percentage of ticks infected per individual host. Hosts were considered infected if they produced >1 infected tick.

## Results

We sampled 3,892 ticks from 214 individual hosts for *B. microti* by real-time PCR (Table 1). We used melting curve analysis and DNA sequencing to confirm efficacy of the real-time PCR. False-positive and false-negative results were rare (Table 2). Of the 79 replicates sequenced with melting point maxima ranging from 83.5°C to 84.5°C, 76 (96.2%) were confirmed as *B. microti* by sequencing. Of the 118 samples sequenced with melting point maxima outside that range, only 2 (1.7%) samples were confirmed as *B. microti* by sequencing. Among samples positive for *B. microti*, 38 of 38 sequences from ticks fed on raccoons (collected from 7 hosts), 3 of 4 sequences from ticks fed on opossums (3 hosts), and 1 sequence from a tick fed on a wood thrush had 2 single bp differences from all other *B. microti*–positive samples sequenced (1 substitution and 1 insertion) (Table 3). One sequence from a tick fed on a raccoon had 1 additional substitution; this sample had a melting point maximum of 82.5°C.

**Table 3:** Sequences of *Babesia microti* 18S rDNA from newly molted nymphal *Ixodes scapularis* ticks fed on vertebrate hosts aligned with sequences from known zoonotic isolates and raccoon isolates from GenBank, southeastern New York, USA, 2008–2010*

Species	Sequence, 5′ → 3′
*Didelphis virginiana* A20030_21	AAGGCAATAACAGGTCTGTGATGCCCTTAGATGTCCTGGGCTGCACGCGCGCTACACTGATG**T**ATTCAACGAG**T**TTTTTCCTTGGC
*Procyon lotor* 506_10	AAGGCAATAACAGGTCTGTGATGCCCTTAGATGTCCTGGGCTGCACGCGCGCTACACTGATG**T**ATTCAACGAG**T**TTTTTCCTTGGC
*Babesia microti* raccoon isolate, USA (AY144701)	AAGGCAATAACAGGTCTGTGATGCCCTTAGATGTCCTGGGCTGCACGCGCGCTACACTGATG**T**ATTCAACGAG**T**TTTTTCCTTGGC
*B. microti* raccoon isolate, Japan (AB197940)	AAGGCAATAACAGGTCTGTGATGCCCTTAGATGTCCTGGGCTGCACGCGCGCTACACTGATG**T**ATTCAACGAG**T**TTTTTCCTTGGC
*Blarina brevicauda* BB3_10	AAGGCAATAACAGGTCTGTGATGCCCTTAGATGTCCTGGGCTGCACGCGCGCTACACTGATG**C**ATTCAACGAG**-**TTTTTCCTTGGC
*Catharus fuscescens,* 2341–01870_17	AAGGCAATAACAGGTCTGTGATGCCCTTAGATGTCCTGGGCTGCACGCGCGCTACACTGATG**C**ATTCAACGAG**-**TTTTTCCTTGGC
*Hylocichla mustelina* 1951–10576_14	AAGGCAATAACAGGTCTGTGATGCCCTTAGATGTCCTGGGCTGCACGCGCGCTACACTGATG**C**ATTCAACGAG**-**TTTTTCCTTGGC
*Peromyscus leucopus* A20003_10	AAGGCAATAACAGGTCTGTGATGCCCTTAGATGTCCTGGGCTGCACGCGCGCTACACTGATG**C**ATTCAACGAG**-**TTTTTCCTTGGC
*Sciurus carolinensis* E412_14	AAGGCAATAACAGGTCTGTGATGCCCTTAGATGTCCTGGGCTGCACGCGCGCTACACTGATG**C**ATTCAACGAG**-**TTTTTCCTTGGC
*Tamias striatus* H1037_11	AAGGCAATAACAGGTCTGTGATGCCCTTAGATGTCCTGGGCTGCACGCGCGCTACACTGATG**C**ATTCAACGAG**-**TTTTTCCTTGGC
*B. microti* GI strain (AF231348)	AAGGCAATAACAGGTCTGTGATGCCCTTAGATGTCCTGGGCTGCACGCGCGCTACACTGATG**C**ATTCAACGAG**-**TTTTTCCTTGGC
*B. microti* Jena/Germany isolate (EF413181)	AAGGCAATAACAGGTCTGTGATGCCCTTAGATGTCCTGGGCTGCACGCGCGCTACACTGATG**C**ATTCAACGAG**-**TTTTTCCTTGGC
*B. microti* Kobe isolate (AB032434)	AAGGCAATAACAGGTCTGTGATGCCCTTAGATGTCCTGGGCTGCACGCGCGCTACACTGATG**C**ATTCAACGAG**-**-TTTTCCTTGGC

*Sequences for zoonotic isolates were obtained from Gray et al. (*4*). The 85–86-bp sequence is the region of 18S rDNA between primers smbaJF and smbaKR. Consistent 2-bp differences between ticks collected from different host species are indicated in **boldface.** The empty row separates sequences with basepair differences. Accessions nos. for GenBank sequences are indicated in parentheses after isolate names. GI, American isolate.

We assessed levels of reservoir competence in 14 host species (10 mammals and 4 birds) (Table 1). White-footed mice, raccoons, short-tailed shrews, and chipmunks had mean levels of reservoir competence >17% (Figure). All other hosts with >10 individuals tested, including opossums, gray and red squirrels, and all 4 species of birds tested (veery [*Catharus fuscescens*], gray catbird [*Dumetella carolinensis*], wood thrush [*Hylocichia mustelina*], and American robin), had mean levels of reservoir competence <6%. Variance in reservoir competence differed significantly among these 11 species (χ^2^ = 73.6973, df = 10, p = 8.525 × 10^−12^, by Fligner-Killeen test of homogeneity of variances). Flying squirrels, striped skunks, and masked shrews all transmitted *B. microti* to ticks, but sample sizes were smaller than those of the other 11 species, leaving us unable to make firm conclusions about relative levels of reservoir competence. There were no host species that did not transmit *B. microti* to any larval ticks (Figure, Table 4). The percentage of hosts infected ranged from 85.7% (raccoons) to 11.8% (robins), and the average percentage of ticks infected by infected hosts ranged from 4.5% (gray catbirds) to 41.8% (white-footed mice), not including host species with a sample size of <10 individuals (Table 4).

**Figure:**
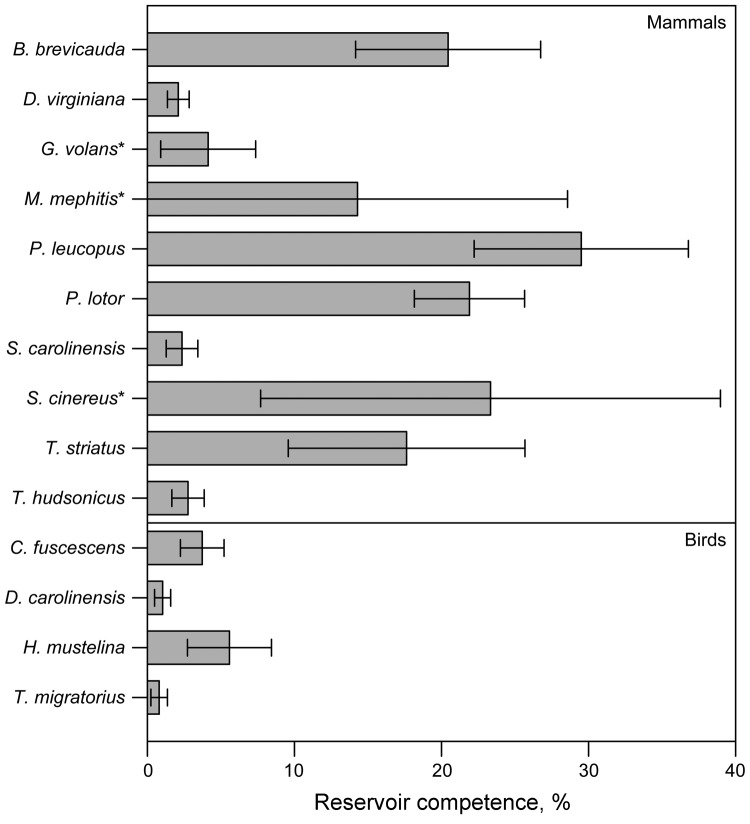
Mean reservoir competence of 14 host species (10 mammals and 4 birds) for *Babesia microti*, southeastern New York, USA, 2008–2010. Error bars indicate SE. Reservoir competence is defined as the mean percentage of ticks infected by any individual host of a given species. Host species with <10 individual hosts sampled are indicated by an asterisk. See Table 1 for sample sizes. Single-letter abbreviations for genera along the left indicate *Blarina*, *Didelphis*, *Glaucomys*, *Mephitis*, *Peromyscus*, *Procyon*, *Sciurus*, *Sorex*, *Tamias*, *Tamiasciurus*, *Catharus*, *Dumetella*, *Hylocichla*, and *Turdus*, respectively.

**Table 4:** Host species infected with *Babesia microti*, southeastern New York, USA, 2008–2010*

Host species	No. (%) infected hosts	No. (%) infected ticks	Mean % infected ticks/infected host (range)
Mammals			
* Blarina brevicauda*	15 (53.6)	103 (19.3)	38.2 (5.6–100.0)
* Didelphis virginiana*	7 (29.2)	10 (2.2)	7.2 (4.3–10.5)
* Glaucomys volans†*	2 (40.0)	2 (2.4)	10.3 (4.0–16.7)
* Mephitis mephitis†*	1 (50.0)	6 (19.4)	28.6
* Peromyscus leucopus*	12 (70.6)	90 (29.2)	41.8 (4.0–90.9)
* Procyon lotor*	18 (85.7)	93 (23.5)	25.6 (4.3–52.6)
* Sciurus carolinensis*	5 (27.8)	9 (2.7)	8.5 (4.2–16.0)
* Sorex cinereus†*	2 (33.3)	12 (29.3)	70.0 (50.0–90.0)
* Tamias striatus*	7 (46.7)	42 (17.1)	37.8 (4.3–90.9)
* Tamiasciurus hudsonicus*	5 (33.3)	9 (3.1)	8.3 (4.0–10.5)
Birds			
* Catharus fuscescens*	6 (40.0)	12 (3.9)	9.3 (4.0–18.8)
* Dumetella carolinensis*	3 (23.1)	3 (1.3)	4.5 (4.2–4.8)
* Hylocichla mustelina*	7 (38.9)	18 (5.7)	14.4 (4.0–50.0)
* Turdus migratorius*	2 (11.8)	2 (0.68)	6.8 (5.3–8.3)

*Infected hosts are those that transmitted *B. microti* to >1 *Ixodes scapularis* tick larvae. For sample sizes, see Table 1. †Host species with <10 individual hosts sampled.

In addition, we tested 414 questing nymphs from 18 field sites for *B. microti* (mean 23 ticks/site, range 12–34 ticks/site). Mean (SD) prevalence of *B. microti* was 16.8% (12.2%). Variation in infection prevalence among sites was high, ranging from 0% to 41.4%. Mean (SD) nymphal density among all sites was 6.49 questing nymphs/100 m^2^ (5.13 questing nymphs/100 m^2^) and ranged from 0.67 to 16.5 nymphs/100 m^2^. On the basis of these data, the mean (SD) density of infected nymphs at these 18 sites was 1.21 nymphs/100 m^2^ (1.62 nymphs/100 m^2^, range 0–6.83 infected nymphs/100 m^2^).

## Discussion

Our broad survey of 14 commonly parasitized, co-occurring mammals and birds in a babesiosis-endemic zone showed variation in *B. microti* reservoir competence among host species. As expected, white-footed mice (*P. leucopus*) act as a competent reservoir for *B. microti,* infecting an average of 29.5% of larval ticks. However, other small mammals (*B. brevicauda* shrews and *T. striatus* chipmunks) and raccoons (*P. lotor*) are also relatively competent reservoirs (Figure), showing mean reservoir competence ranging from 17.6% to 21.9%. All other wildlife species with >10 individuals tested had low levels of *B. microti* transmission; no individual host species infected >6% of ticks on average. Depending on their tick loads and relative abundances, these species have the potential to lower rates of disease risk (i.e., infection prevalence) by feeding ticks but not transmitting this pathogen (*35*). Within a host species, individual differences such as age, reproductive status, sex, time since infection, and degree of co-infection may affect transmission of *B. microti*. Our approach focused on sampling a breadth of species under a range of natural conditions rather than sampling any single species in sufficient depth to take potential causes of intraspecific variation into account. Future studies examining the mechanisms controlling individual variation in reservoir capacity are warranted because we observed considerable variation in reservoir capacity between individual species (Table 4).

The average level of *B. microti* infection in questing nymphal ticks was intermediate between more and less competent reservoir species, consistent with the fact that larval ticks are feeding on a combination of hosts with varying levels of reservoir competence. Variation in *B. microti* prevalence in questing ticks among sites could reflect differences in host community composition or differences in infection prevalence among hosts at different sites. Site-specific differences in the density of questing nymphs at each site also contribute to variation in the density of infected nymphs among sites. Nymphal densities measured fall within the range documented in previous studies (*36*). Future studies should focus on sampling questing nymphs more broadly across landscapes to link levels of infection in questing ticks and the density of infected nymphs to host communities and other landscape-scale habitat variation. Monitoring of babesiosis-endemic areas for temporal changes in nymphal infection prevalence may also help in understanding mechanisms for the regional increase in human babesiosis cases.

Ground-dwelling bird species have not been sampled frequently for their capacity to transmit *B. microti* to ticks, but they might play a role in disease transmission and spread because they are exposed to *I. scapularis* ticks. Of the 4 species tested in this study, wood thrushes (*H. mustelina*) had the highest reservoir competence of all birds tested (5.6%), but the other 3 bird species were also able to transmit the pathogen, albeit at low levels (<4%). Transmission of *B. microti* has been detected in other bird species; positive *I. ricinus* tick larvae have been found feeding on European robins (*Erithacus rubecula*) (*32*) and other birds (*30*,*31*).

The real-time PCR developed for this study was effective in amplifying *B. microti* DNA and had a high level of specificity for this pathogen. The measured rates of false-positive and false-negative results were <5% (Table 2). The low false-positive rate is dependent on melting curve analysis after amplification. False-negative results tended to occur at melting point maxima just below what we considered diagnostic for *B. microti* (82.5°C–83°C), and may represent strain variation within *B. microti*. In future studies, it may be necessary to directly sequence samples with these melting point maxima.

On the basis of the short fragments that we sequenced, our samples of *B. microti* suggest some degree of host specialization (Table 3), consistent with previous work showing divergent clades of *B. microti* (*37*,*39*,*40*). Variation found in raccoon and opossum isolates matches variation in other raccoon isolates (GenBank accession nos. AY144701 and AB197940). We stress the preliminary nature of this result given the short sequence length and the relative paucity of *B. microti* sequences available in GenBank. The role of raccoons and opossums in human babesiosis dynamics depends on the zoonotic potential of the *B. microti* strains they carry. Phylogenetic analyses of 18S and β-tubulin genes (*37*) and the chaperonin-containing t-complex polypeptide l gene (*39*) of *B. microti* showed a distinction between raccoon isolates and known zoonotic strains. Given that all host species sampled transmitted *B. microti*, further studies detailing the level of genetic differentiation between *B. microti* samples isolated from different hosts are critical, as is a thorough assessment of the genetic diversity of *B. microti* infections of humans. Improved understanding of *B. microti* strain diversity is necessary to consider the public health implications of the role of different hosts in *B. microti* dynamics.

In this study, we demonstrated that *P. leucopus* mice are a competent reservoir host for *B. microti*, but other small mammals and raccoons have comparable reservoir competence and might play a critical role in disease transmission, depending on their tick loads and relative abundance (*34*,*35*). At least 1 individual of all wildlife host species sampled transmitted *B. microti* to >1 *I. scapularis* tick, even when host sample sizes were relatively low. Tick vectors that transmit this pathogen interact with strongly and weakly competent reservoir hosts, and this variation in reservoir competence within host communities should be considered when predicting risk for infection with *B. microti* based on animal community composition. To explain the recent emergence of human babesiosis, the community ecology of *B. microti* needs to be understood in greater depth.
